# A critical pedagogy approach to reimagining professionalism in clinical associate/clinical officer training in Sub-Saharan Africa

**DOI:** 10.3389/fmed.2026.1983787

**Published:** 2026-10-15

**Authors:** Esther Cele, Nabeela Sujee

**Affiliations:** 1 Unit for Undergraduate Medical Education (UUME), School of Clinical Medicine, Faculty of Health Sciences, University of the Witwatersrand, Johannesburg, South Africa; 2 Centre for Health Science Education, Faculty of Health Sciences, University of Witwatersrand, Johannesburg, South Africa

**Keywords:** clinical associate, clinical officers, critical pedagogy, health profession education, professionalism, Sub-Saharan Africa, Ubuntu

## Abstract

Clinical Associates and Clinical Officers (CA/COs) play an increasingly important role in strengthening primary healthcare systems across Sub-Saharan Africa, particularly in underserved and under-resourced communities. Despite their community-oriented mandate, professionalism within health professions education continues to be predominantly conceptualised through biomedical, competency-based, and individualised frameworks that prioritise observable behaviours and professional attributes. While valuable, these approaches may inadequately account for the historical, social, political, and relational dimensions of practice that shape healthcare delivery in contexts marked by colonial legacies, health inequities, and workforce shortages. This paper aims to explore how critical pedagogy can be used to critique and reimagine professionalism within CA/CO education in Sub-Saharan Africa. Drawing on the work of Paulo Freire, alongside scholarship from health professions education, decolonial theory, and professionalism studies, we undertake a conceptual analysis of dominant professionalism discourses and examine their applicability to the realities of CA/CO practice. Through the Freirean concepts of dialogue, praxis, and critical consciousness, as well as drawing from concepts of Ubuntu we propose an alternative conceptualisation of professionalism that is relational, contextual, political, and emancipatory. We argue that critical professionalism as a reimagined approach should extend beyond adherence to predefined competencies and behaviours towards a critically informed engagement with communities, health systems, and the structural conditions that influence health and healthcare. By foregrounding social accountability, equity, and contextually responsive practice, critical pedagogy offers a theoretically grounded approach for reimagining professionalism within CA/CO education. This perspective has implications for curriculum design, faculty development, professionalism assessment, and professional identity formation, whilst contributing to broader discussions regarding socially responsive and decolonially informed health professions education.

## Introduction

In rural and underserved areas of Sub-Saharan Africa, where physician shortages persist, Clinical Associates/Clinical Officers play a crucial role in delivering primary healthcare by expanding access, easing health system strain, and bringing services closer to communities. Professionalism is usually defined as a mix of values, behaviours, and moral responsibilities guiding interactions with patients, colleagues, and systems, but dominant biomedical, rule-based frameworks focus on individual attributes and standardised competencies, and thus inadequately address power dynamics, colonial legacies, and the obligation of mid-level cadres to marginalised populations. This paper offers a conceptual and theoretically informed approach which argues that professionalism in CA/ CO training demands more than replicating generic biomedical ideals. It requires critical engagement attuned to sub-Saharan Africa's unequal health systems. Drawing on health professions education research and justice-oriented, decolonial critiques of curriculum, it explores how critical pedagogy can surface, critique, and reimagine professionalism to foster relational practices, social accountability, and ethical identity formation in CA/CO roles. This paper is a conceptual contribution to existing discussions of professionalism in health professions education. First, it applies critical pedagogy to the relatively underexplored area of professionalism formation among CAs and COs in sub-Saharan Africa, two cadres that play important roles in frontline healthcare yet remain largely absent from the professionalism literature. Second, it brings Freirean ideas of dialogue, praxis and critical consciousness into conversation with Ubuntu, highlighting how professionalism is shaped through both critical engagement with social realities and relationships with others. Third, it offers a conceptual framework that positions professionalism as a socially situated process informed by context, relationships and a commitment to social accountability.

## Background and context

Healthcare in Sub-Saharan Africa has been shaped by diverse social, cultural, and historical influences. Prior to colonialism, health and healing were largely embedded within communities and indigenous knowledge systems, where care was closely connected to social relationships, cultural practices, and local understandings of wellbeing. Colonialism reconstructed health services in ways that privileged particular forms of knowledge, authority, and professional practice, reshaped healthcare delivery and established hierarchies that continue to influence contemporary health systems (1, 2).

The development of primary health care (PHC) in post-colonial systems placed greater emphasis on community participation, equity, accessibility (1, 2). The training of community health workers was an effort to provide healthcare services to communities previously and commonly marginalised as a result of colonialism and, in some countries, Apartheid systems (2, 3).

This history is important for understanding contemporary healthcare practice and professionalism in Sub-Saharan Africa. As a result, professionalism cannot be understood solely through decontextualised biomedical frameworks but must be considered in relation to the social, historical, and community realities within which healthcare is delivered (1, 2). It is within this context that Clinical Associates and Clinical Officers (CA/CO's) emerged as key cadres designed to strengthen access to care and support primary healthcare delivery in underserved communities (3).

CA/CO's originated in East Africa as COs, with Uganda establishing the cadre in 1918 and Kenya following in 1928 to address healthcare shortages, particularly in rural areas. The model subsequently evolved and gained formal recognition with countries including Malawi, Tanzania, Zambia, and South Africa developing their own CA training programme (4). These practitioners are being integrated and scaled up as targeted workforce strategy to enhance access to essential clinical care, alleviate the burden on strained health systems, and bring PHC services closer to local communities (3). The training of these cadres focused on preparing them for collaborative roles within multidisciplinary healthcare teams ensuring holistic patient care (5, 6). Central to the profession's purpose is the development of a community- and person-centred healthcare practitioner who is practically oriented, skilled in hands-on clinical care, and able to make a meaningful contribution to improving health system performance and patient outcomes (3).

While CAs and COs do not constitute a uniform profession across sub-Saharan Africa, they share several historical, workforce and health service characteristics that warrant considering their professionalism needs in relation to one another. COs have an established presence in many East, Central and South African countries, where they fulfil diverse clinical functions that may include patient assessment, diagnosis, treatment, emergency care and, in some contexts, surgical and procedural duties (7–9). In South Africa, by comparison, CAs complete a three-year bachelor's degree and are primarily employed within district health services, practising under the oversight of medical practitioners in accordance with regulations set by the Health Professions Council of South Africa (HPCSA) (10, 11). Consequently, educational preparation, professional recognition, scope of practice and relationships with medical practitioners vary considerably across settings. Despite these differences, both cadres occupy mid-level clinical positions within multidisciplinary healthcare teams and frequently serve as a bridge between communities and formal health services. In doing so, they often negotiate entrenched professional hierarchies while addressing complex and contextually grounded health needs (3, 11).

Although the day-to-day responsibilities of CAs and COs are not identical, both cadres practice within health systems marked by constrained resources, persistent workforce gaps, considerable community health demands and deeply embedded professional hierarchies (12). These shared realities give rise to comparable tensions in the development of professional identities that are attuned to social accountability, sensitive to inequities and committed to the wellbeing of the communities they serve. Considering CA and CO professionalism alongside one another therefore creates an opportunity to examine how critical pedagogy might foster professional formation in distinct yet similarly positioned frontline healthcare settings.

The literature suggests that these mid-level roles effectively address workforce shortages, particularly with adequate oversight, tailored skills-training, and retention strategies (7). However, the implementation of task shifting is not neutral, as it has sparked challenges around role clarity, accountability, and professional limitations, significantly impacting how professional identity takes shape in nuanced ways (3, 13). For CA/CO students, professional identity forms through both structured coursework and experiential learning, where interactions with supervisors and allied healthcare professionals can enable recognition and deepen the sense of belonging, or conversely, reinforce marginalisation within team hierarchies. As professional identity develops through both personal and contextual influences, learners simultaneously construct understandings of what it means to be a professional and which values, behaviours, and responsibilities are associated with professional practice (3). Professionalism is typically viewed as a blend of core values, actions and moral duties that shape how health workers engage with patients, peers, and the broader system (13). However, leading frameworks for professionalism often draw from biomedical and normative perspective, prioritizing individual attributes, standardized competencies, and rule-based ethics (13).

CA/CO's have a set of professional competencies and attributes which differ based on country and community-needs, however, the alignment to meet these and the current training models are often mismatched. The attributes mentioned above are often adapted from well-recognised competency frameworks such as CANMED's, ACGME, AACN (14) which include the following: medical expert, communicator, collaborator, leader, health advocate, scholar and professional. In South Africa, the National Department of Health has adopted Entrustable Professional Activities (EPAs) as exit outcomes for Clinical Associates, encompassing patient assessment and comprehensive management, health promotion, communication and collaboration, improvement of healthcare services, and professional development, including the requirements to “practice professionalism” and “practice ethically” (14). However, professionalism is not simply a neutral collection of universally applicable attributes; it is constructed through professional values, behaviours and expectations that are taught, modelled and assessed within health sciences education. Kirk describes professionalism in terms of both underlying values and observable behaviours, highlighting the importance of how these behaviours are defined and communicated to learners (15). This raises important questions about whose values and ways of being are privileged in defining what constitutes a “professional” healthcare practitioner, particularly within historically colonial and culturally diverse contexts such as sub-Saharan Africa. Although many of the current curricula explicitly recognise the importance of understanding patients within their family and community contexts and acknowledge indigenous forms of and approaches to healing, specifically traditional healers, within the healthcare system (1, 2). The broader conceptualisation and enactment of professionalism is still shaped by dominant biomedical and historically inherited professional norms. Consequently, educators have an important role not only in helping students understand and enact established expectations of professionalism, but also in critically examining the assumptions, power relations and cultural norms underpinning these expectations. Without creating opportunities for learners to question and critically engage with these structures, professional education may inadevertently reproduce existing hierarchies and norms rather than enabling students to develop forms of professionalism that are both ethically rigorous and responsive to the sociocultural contexts in which healthcare is practiced. Critical pedagogy provides a way to reimagine professionalism and how teaching it in a contextually grounded manner can build thoughtful, community-focused professionals.

## Critical pedagogy as a transformative lens

The limitations of dominant professionalism frameworks become particularly apparent when considered in relation to the realities of Clinical Associate and Clinical Officer (CA/CO) practice in Sub-Saharan Africa. As discussed above, prevailing conceptions of professionalism in health professions education tend to emphasise individual attributes, prescribed behaviours, and adherence to established professional norms. While these approaches provide important guidance for professional conduct, they often position professionalism as a largely personal achievement detached from the broader social, historical, and political contexts in which healthcare is delivered (16–18). Such framings inadequately account for the realities of CA/CO practice, which is inherently community-oriented, relationship-centred, and deeply embedded within health systems marked by resource constraints, professional hierarchies, and enduring social inequities (8, 19).

Critical pedagogy offers a powerful framework through which such a reconceptualisation can occur. Rooted in the work of Paulo Freire, critical pedagogy views education as a political, relational, and emancipatory endeavour rather than a neutral process of transmitting knowledge (20). Freire rejected educational approaches that position learners as passive recipients of predetermined truths and instead advocated for educational practices that enable learners to interrogate taken-for-granted assumptions, question existing power structures, and actively participate in shaping their world (20).

## Re-imagining professionalism through critical pedagogy

Central to the reimagining are Freire's concepts of dialogue, praxis and critical consciousness (20, 21). Dialogue challenges hierarchical assumptions about who holds knowledge and whose perspectives matter (20). Through dialogue, educators and learners co-construct understanding, engage with multiple perspectives, and critically examine the assumptions that underpin professional practice (20). In CA/CO education, dialogic learning creates opportunities to explore tensions surrounding authority, role legitimacy, interprofessional relationships, and responsibilities toward communities. Professionalism therefore emerges not solely from individual conduct, but through relationships with patients, colleagues, communities, and healthcare systems (18). It becomes grounded in reciprocity, humility, mutual respect, and attentiveness to the experiences of others (16, 22).

Freire's notion of praxis further extends professionalism beyond reflection towards action (20, 21). Praxis describes the continual interplay between critical reflection and purposeful action that seeks to transform the conditions giving rise to injustice (21). For CA/COs, this means recognising how health inequities, resource constraints, professional hierarchies, and historical legacies influence healthcare delivery, while also considering how professional practice can contribute to meaningful change. Professionalism is therefore not limited to competent clinical performance but encompasses social accountability, advocacy, and responsiveness to community needs (16, 23). Through praxis, learners begin to see themselves not only as healthcare providers, but as participants in strengthening health systems and advancing more equitable forms of care.

The development of critical consciousness (conscientisation), enables learners to become aware of the social, political, historical, and structural forces that shape professional practice (20, 21, 24). For CA/COs, this includes recognising how colonial histories, professional hierarchies, workforce shortages, and inequitable resource distribution have influenced both healthcare delivery and professional identity formation. Awareness of these factors encourages learners to see professionalism not simply as conformity to established standards, but as a reflective and ethical engagement with the conditions that shape practice (20, 24). Critical pedagogy shifts attention from what professionals should do towards examining why certain professional norms exist, whose interests they serve, and how they might be transformed to better respond to the needs of patients, communities, and health systems (16, 20, 21).

Building on these concepts, we propose critical professionalism as a form of professional practice which is characterised by critical consciousness, reflexivity, accountability and attentiveness to the conditions that influence health and healthcare (16, 22). We present it as a possible outcome of critical pedagogical approaches to professional formation, with relevance for both practitioners and the institutions in which they learn and work. Figure 1 outlines this proposed approach.

**Figure 1:**
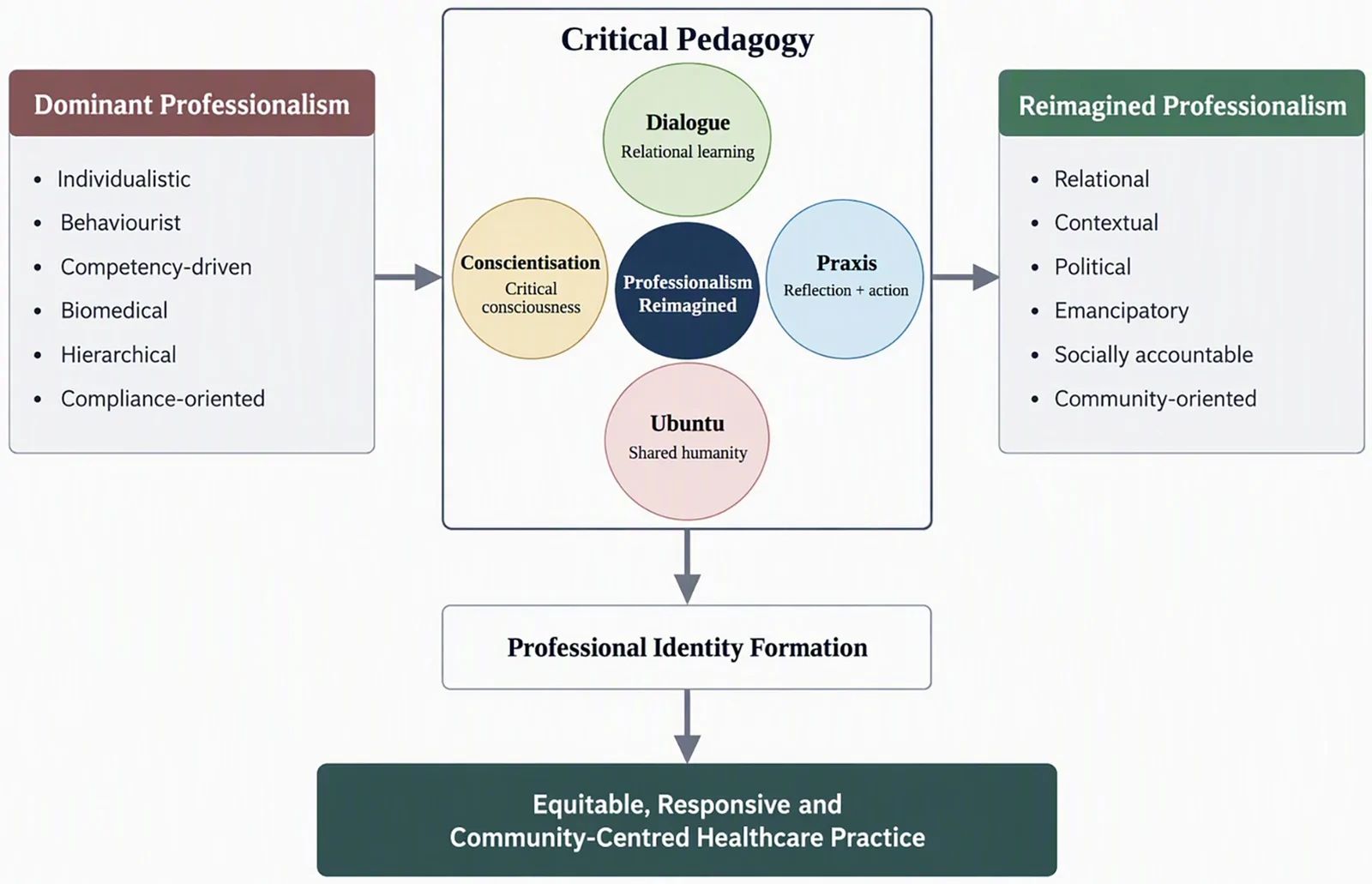
Conceptual image of a re-imagined critical professionalism in CA/CO training.

An additional consideration in rethinking professionalism is the contribution of community perspectives to defining what professional practice should involve (25, 26). A community-responsive understanding of professionalism requires moving beyond seeing patients and communities only as recipients of care and recognising them as important contributors to professional formation and knowledge generation. Consistent with the principles of critical pedagogy, community members, traditional healers, local health workers and other relevant stakeholders can help shape understandings of professional practice through participation in curriculum development, discussions about local health priorities, community-based learning activities and feedback processes (27). However, community perspectives are diverse across sub-Saharan Africa, and careful attention is needed to whose voices are included, whose may be overlooked and how different views are engaged. Communities can provide valuable insights into important relational aspects of professionalism, such as respect, trust, responsiveness, cultural humility, accountability and commitment to local priorities.

Viewed in this way, professionalism becomes inherently contextual and political. Its meaning cannot be separated from the settings in which care is delivered or from the social, cultural, historical, and economic forces that shape those settings (13).

Taken together, dialogue, praxis, critical consciousness point towards a professionalism that is relational, contextual, political, and emancipatory. In this formulation, professionalism is no longer understood simply as adherence to prescribed behaviours or competencies. Rather, it becomes an ongoing process of engaging ethically and critically with communities, health systems, and the broader social conditions that shape health and healthcare. For CA/COs, whose work is situated at the intersection of community needs and health system realities, such a conception offers a more contextually relevant and socially responsive foundation for professional identity formation.

Implications for practiceUbuntu provides an important lens through which professionalism can be understood within sub-Saharan African healthcare settings (25, 28). Grounded in the African philosophy often expressed through the phrase *umuntu ngumuntu ngabantu* (“a person is a person through other people”), Ubuntu views personhood, humanity and moral growth as developing through relationships, interdependence and community (29). From this perspective, professionalism is shaped by individual knowledge and skills and equally by relationships with patients, communities, colleagues and society. Ubuntu therefore draws attention to values such as interconnectedness, mutual responsibility and commitment to others, offering a contextually relevant way of understanding professional identity and practice.

Contemporary approaches to professionalism increasingly recognise that professional practice involves more than technical competence and includes ethical, social and relational dimensions (13). A critical pedogeological perspective situates competency-based approaches within the realities of healthcare practice (27, 30). Competencies, professional behaviours and EPAs remain important; however, they may be limited when viewed in isolation from the social and institutional conditions in which care is provided. Critical pedagogy therefore encourages reflection on how professionalism is shaped by context, including issues of power, inequality, resource availability and community priorities (31). Through this lens, Ubuntu can complement existing professionalism frameworks by supporting a more socially accountable and contextually grounded understanding of professional formation and practice.

### Assessing critical professionalism

Educators need to move beyond viewing professionalism primarily as a set of behaviours to be demonstrated and assessed and instead create opportunities for learners to reflect on their experiences, engage in meaningful dialogue and examine the realities of the communities they serve (20, 32). A critical professionalism approach may involve reflective narratives, community-engaged learning, structured dialogue, medical humanities and simulation activities that explore ethical tensions, power relations, social inequities and team dynamics alongside clinical competence (33). Faculty development is important to equip educators to facilitate these learning experiences and to model forms of professionalism that are responsive to context and grounded in social accountability. At the same time, curriculum developers, institutions and regulatory bodies should reconsider how professionalism is defined, taught and assessed to ensure that engagement with communities and responsiveness to local realities are recognised as important aspects of professional practice (26).

Assessment should also move beyond reliance on checklists and prescribed behaviours. Attributes such as critical consciousness, reflexivity, accountability and social responsiveness develop over time and are shaped by context. In critical professionalism, the focus should therefore be on learners’ growing ability to reflect on their practice, engage with different perspectives, recognise the factors that influence health and healthcare, and respond appropriately within their professional settings. Consistent with programmatic assessment, this requires judgement based on multiple sources of evidence gathered over time, capturing learning, reflection and growth alongside demonstrated competence (34). Input from educator's supervisors, peers, patients, and where appropriate, community members can contribute to a more complete understanding of professionalism, recognising that it is developed and expressed through relationships and is best assessed from multiple viewpoints. Community perspectives should complement professional and educational judgement.

### Institutional, faculty and learner conditions for critical professionalism

A key challenge for critical professionalism is that encouraging learners to question professional hierarchies, institutional practices and inequities may place them at odds with the settings in which they learn and work (35, 36). This may be particularly relevant for CAs and COs, whose roles may already be subject to uncertainty or contestation within some healthcare environments (11). For critical pedagogy to be meaningful, institutions must create conditions in which questions and concerns can be raised and discussed constructively without being treated as a challenge to authority. At the same time, health professions education has a responsibility to uphold patient safety, ethical practice and professional standards. Navigating this tension requires recognising the difference between core professional obligations and the ways in which these are interpreted and enacted within contexts. In this way, critical engagement strengthens professional accountability by encouraging reflection on how professional standards are applied in practice. Preparing for the implementation of critical pedagogy therefore requires commitment from both learners and institutions, with organisations willing to engage with constructive critique while maintaining safe and ethical practice.

## Conclusion

Clinical Associates and Clinical Officers occupy a distinctive and increasingly important position within Sub-Saharan African health systems, working at the intersection of communities, healthcare institutions, and multidisciplinary teams. Yet dominant professionalism frameworks remain largely rooted in biomedical, individualistic, and behaviourist traditions that insufficiently account for the contextual, relational, and historical realities shaping their practice. Drawing on Freire's critical pedagogy, this paper has argued for critical professionalism as an approach that moves beyond compliance with prescribed norms towards a more critical, relational, and socially responsive understanding of professional identity.

Through critical consciousness, dialogue, and praxis, critical professionalism becomes a process of engaging ethically with patients, communities, colleagues, and health systems while recognising and addressing the structural conditions that influence health and healthcare. The framework extends competency-based approaches by recognising that professional practice is shaped by individual competence and by the communities, health systems and broader conditions within which healthcare is delivered. Although critical pedagogy, decolonial perspectives, social accountability and professionalism have each been discussed within health professions education, they have seldom been considered together in relation to the professional formation of CAs and COs in sub-Saharan Africa (16, 26, 31). Grounded in the context of Sub-Saharan Africa and attentive to the legacies of coloniality and inequality, this vision positions professionalism as relational, contextual, political, and emancipatory. Such a perspective offers a theoretically grounded and contextually relevant foundation for CA/CO education and provides a pathway towards developing health professionals who are not only clinically competent, but also socially accountable, critically aware, and committed to advancing more equitable and humane healthcare systems.
